# New insight into the metabolic mechanism of a novel lipid-utilizing and denitrifying bacterium capable of simultaneous removal of nitrogen and grease through transcriptome analysis

**DOI:** 10.3389/fmicb.2023.1258003

**Published:** 2023-10-30

**Authors:** Yaobin Tong, Yiyi Li, Wenpan Qin, Shengchun Wu, Weiping Xu, Peng Jin, Zhanwang Zheng

**Affiliations:** ^1^School of Environmental & Resource, Zhejiang A & F University, Hangzhou, China; ^2^Zhejiang Sunda Public Environmental Protection Co., Ltd., Hangzhou, China; ^3^College of Food and Health, Zhejiang A & F University, Hangzhou, China

**Keywords:** grease wastewater, carbon source, denitrifying bacteria, transcriptome, metabolism pathways

## Abstract

**Introduction:**

Issues related to fat, oil, and grease from kitchen waste (KFOG) in lipid-containing wastewater are intensifying globally. We reported a novel denitrifying bacterium *Pseudomonas* CYCN-C with lipid-utilizing activity and high nitrogen-removal efficiency. The aim of the present study was aim to explore the metabolic mechanism of the simultaneous lipid-utilizing and denitrifying bacterium CYCN-C at transcriptome level.

**Methods:**

We comparatively investigated the cell-growth and nitrogen-removal performances of newly reported *Pseudomonas glycinae* CYCN-C under defined cultivation conditions. Transcriptome analysis was further used to investigate all pathway genes involved in nitrogen metabolism, lipid degradation and utilization, and cell growth at mRNA levels.

**Results:**

CYCN-C could directly use fat, oil, and grease from kitchen waste (KFOG) as carbon source with TN removal efficiency of 73.5%, significantly higher than that (60.9%) with sodium acetate. The change levels of genes under defined KFOG and sodium acetate were analyzed by transcriptome sequencing. Results showed that genes *cyo*, *CsrA*, *PHAs*, and *FumC* involved in carbon metabolism under KFOG were significantly upregulated by 6.9, 0.7, 26.0, and 19.0-folds, respectively. The genes *lipA*, *lipB*, *glpD*, and *glpK* of lipid metabolic pathway were upregulated by 0.6, 0.4, 21.5, and 1.3-folds, respectively. KFOG also improved the denitrification efficiency by inducing the expression of the genes *nar*, *nirB*, *nirD*, and *norR* of denitrification pathways.

**Conclusion:**

In summary, this work firstly provides valuable insights into the genes expression of lipid-utilizing and denitrifying bacterium, and provides a new approach for sewage treatment with reuse of KFOG wastes.

## 1. Introduction

Issues related to fat, oil, and grease from kitchen (KFOG) waste in sewer systems are intensifying on a globally scale. Restaurant lipid-containing wastewater comprises fatty acids, triacylglycerols, and lipid soluble hydrocarbons, which are the main cause of the high chemical oxygen demand (COD) in lipid-containing wastewater (Ahmad et al., 2022). These KFOG blockages are difficult to hydrolyze naturally, leading to the overflow of sanitary sewers and sewage contamination of water bodies. Excess KFOG in lipid-containing wastewater inhibits the growth, lipid-utilization efficiency and nitrogen-removal ability of bacteria. The self-repair capacity of water environment is also reduced, thereby worsening the water quality. Over the last few years, the comprehensive utilization of kitchen waste as a carbon source has attracted widespread attention. The fermentation broth of kitchen waste contains glycerol and volatile fatty acids, which are a good source of microbial carbon (Kim et al., 2016). Some scholars explored the ability of single vegetable oil or animal oil as a carbon source (Ettayebi et al., 2003; Hasanuzzaman et al., 2004); however, the potential of KFOG, as a carbon source has not been verified. The collection and treatment of KFOG are time consuming and costly, thereby limiting its industrial application.

The emission standards of domestic wastewater in China has recently increased and become stricter. According to the “Discharge standard of pollutants for municipal wastewater treatment plant” (GB 18918-2002) in China, the first-level standard A specifies that the maximum allowable limit for municipal wastewater discharge should not exceed 15 mg/L of total nitrogen (TN), 5 mg/L of NH_4_^+^-N, 50 mg/L COD, and 1 mg/L of animal and plant oils. Biological nitrogen removal has attracted widespread attention in wastewater treatment owing to its efficient and low-cost nitrogen removal from water in the form of biological nitrogen and N_2_ through the assimilation, nitrification, and denitrification of denitrifying microorganisms (Ding et al., 2023). Many aerobic denitrifying bacteria, such as *Ochrobactrum anthropic* HND19 (Ren et al., 2021), *Enterobacter* sp. HNDS-6 (Liu et al., 2023), and *Pseudomonas psychrophile* HA-2 (Luo et al., 2022) have been acquired from aquaculture environments. However, most denitrifying bacteria are heterotrophic, and denitrification requires sufficient carbon sources to provide energy and electrons. Unfortunately, the low carbon-to-nitrogen (C/N) ratio of domestic wastewater seriously compromise the nitrogen-removal efficiency of biological treatment by denitrifying bacteria. Adding carbon sources is the main way to improve the treatment efficiency, but this process translates into heavy costs. The use of alternative, green, and low-cost carbon sources for wastewater treatment exhibits great potential. Many lipase-producing bacteria, such as *Thalassospira permensis* M35-15 (Isiaka Adetunji and Olufolahan Olaniran, 2018), *Bacillus* sp. B304 (Mongkolthanaruk and Dharmsthiti, 2002), *Pseudomonas mosensis* TB5 (Sahoo et al., 2021), and *Acinetobacter baumannii* RMUTT3S8-2 (Bunmadee et al., 2022), grow in aquaculture environments. They could metabolize and grow well with lipids as the single carbon source. However, the few reports on denitrifying bacteria that use KFOG as carbon source for growth and denitrification limit the *in-situ* utilization of this waste in lipid-containing wastewater treatment. The nitrogen metabolism of denitrifying bacteria involves multiple pathways, such as nitrification (NH4^+^-N to NO_3_^−^-N/NO_2_^−^-N), denitrification (NO_3_^−^-N/NO_2_^−^-N to N_2_), and assimilation (NH4^+^-N to biological nitrogen) (Hou et al., 2021). The effect of the nitrogen-removal ability of lipid-utilizing bacteria on lipid utilization is seldom investigated. Understanding the nitrogen-removal mechanism of lipid-degrading and denitrifying bacteria is essential.

In this study, a novel bacterium named *Pseudomonas* CYCN-C capable of simultaneous lipid utilization and denitrification was screened from kitchen sewer. CYCN-C could use KFOG as carbon source for lipid degradation and efficient nitrogen removal. We investigated the performance of this bacterium for various C/N, temperatures, and lipids. We further comparatively analyzed all pathway genes involved in nitrogen metabolism, lipid degradation and utilization, and cell growth at the transcriptomic levels under culture conditions with different carbon sources. This work contributed to the understanding of the mechanism underlying the simultaneous lipid metabolism and nitrogen-removal of bacteria at the gene metabolism levels.

## 2. Materials and methods

### 2.1. Isolated and media

The bacterium CYCN-C was isolated from a kitchen sewer sewage sample, and KFOG was obtained from the same sample. Approximately 1 mL of sewer sewage was added to 100 mL of sterile deionized water, incubated at 30°C for 1 h, and diluted to 10^−5^-fold. The sample was inoculated on nitrification medium (NM). A batch of bacteria with efficient nitrogen-removal ability was obtained through shake-flask experiments. Each isolated strain was inoculated into the lipase-production medium plates and cultivated at 30°C for 24 h. A bacterium with lipolytic activity was selected from the lipase-production medium plates and named CYCN-C for further studies.

The NM comprised 5.0 g of sodium acetate, 0.235 g of (NH_4_)_2_SO_4_, 0.5 g of KH_2_PO_4_, 0.5 g of Na_2_HPO_4_, 0.4 g of MgSO_4_·7H_2_O, and 2.00 mL of trace elements per liter (initial pH of 7). The lipase-production medium comprised 1% olive-oil emulsion, 5.0 g of yeast extract, 10.0 g of peptone, and 5.0 g of NaCl per liter (pH 7). An olive-oil emulsion was obtained by mixing 4% polyvinyl alcohol with soybean oil at a ratio of 3:1 and treating it for 6 min in a high-speed homogenizer. The synthetic wastewater medium (SWM) comprised 2.0 g of olive oil, 0.235 g of (NH_4_)_2_SO_4_, 0.5 g of KH_2_PO_4_, 0.5 g of Na_2_HPO_4_, and 0.4 g of MgSO_4_·7H_2_O, and 2.00 mL of trace elements per liter (initial pH of 7) (Matsumiya et al., 2007). The trace-element solution contained 50.0 g of EDTA-2Na, 2.2 g of ZnSO_4_, 5.5 g of CaCl_2_, 5.06 g of MnCl_2_·4H_2_O, 5.0 g of FeSO_4_·7H_2_O, and 1.6 g of CoCl_2_·6H_2_O per liter (pH 7). Each medium was sterilized at 121°C for 30 min, and a solid medium of 2% agar was added.

### 2.2. Identification of strain 16SrDNA

Single colonies were selected for colony PCR, and the primers used were 27F (5′-AGAGTTTGATCCTGGCTCAG-3′) and 1495R (5′-CTACGGCTACCTTGT TACGA-3′). PCR was carried out as follows: 5 min at 94°C, 30 cycles of 30 s at 94°C, 30 s at 55°C, 1.5 min at 72°C, and a final step of 10 min at 72°C. The PCR products were purified and sequenced by Zhejiang Shangya Biotechnology Co., Ltd., China. The obtained sequences were retrieved and compared by Basic Local Alignment Search Tool (BLAST) in GenBank,^1^ and 16 representative bacterial 16S rDNA sequences with high similarity to this sequence were screened out. A phylogenetic tree was constructed with MEGA7.0 to search for the strains closest to its homology.

### 2.3. Performance verification of CYCN-C

#### 2.3.1. Assessment of denitrification performances

CYCN-C were cultivated in NM at 30°C with shaking at 180 rpm for 24 h, and then used as seed cultures (approximately 4 × 10^8^ CFU/mL). For the verification of the influence of different carbon sources on nitrogen removal efficiency, 300 mL of the NM with KFOG (15 g/L), sodium acetate (5 g/L), sodium citrate (5 g/L), or sodium succinate (5 g/L) as the single carbon source was inoculated with 3 mL of CYCN-C seed culture and then cultured at 30°C with shaking at 180 rpm. For the C/N ratio experiments, the C/N ratios were set as 1, 3, 5, 7, and 10. Sampling was regularly performed to measure the OD_600_，NH4^+^-N, and TN. All the experiments were performed in triplicate.

#### 2.3.2. Assessment of lipid-utilization capabilities

CYCN-C were cultivated in SWM at 30°C with shaking at 180 rpm for 24 h, and then used as seed cultures. In brief, 100 mL of SWM was inoculated with 1.0 mL of CYCN-C seed culture and then cultured at 30°C with shaking at 180 rpm for 48 h. The factors influencing the lipid utilization efficiency of CYCN-C, including lipid concentration and temperature, were analyzed by separate experiments. For the lipid-concentration experiments, the lipid concentrations were set to 1, 2, 3, 4, and 5 g/L. For the temperature experiments, the culture temperatures were 10°C, 20°C, 30°C, 40°C, and 50°C. Sampling was regularly performed to measure the lipid concentration. All the experiments were performed in triplicate. The performance verification results were statistically analyzed by one-way ANOVA using IBM SPSS Statistics 27 software.

### 2.4. Transcriptome analysis

#### 2.4.1. Total RNA extraction

Transcriptome analysis was conducted to quantify the messenger RNA (mRNA) levels during cell metabolism. Bacterial samples were collected from the NM with KFOG or sodium acetate as single carbon sources in the middle of the logarithmic growth stage (OD_600_ = 1.0). The collected cells were quickly frozen in liquid nitrogen. The total mRNA of strains was extracted using a Bacterial Total RNA extraction kit (Hangzhou Biosci Co., Ltd., China) in accordance with the manufacturer’s specifications. RNA degradation and contamination were monitored on 1% agarose gels. RNA integrity and total RNA were accurately detected using Agilent 2,100 bioanalyzer (Agilent Technologies, CA, United States).

#### 2.4.2. Transcriptome library preparation and sequencing

After the preparation of high-quality RNA, a cDNA library was constructed according to chain-specific fragmentation. The first strand of cDNA was synthesized using fragmented mRNA as a template and random oligonucleotides as a primer in the M-MuLV reverse transcriptase system. The synthesized first strand was then degraded by RNaseH, and the second strand of cDNA was synthesized by DNA polymerase I. The cDNA was screened for a size range of 370–420 bp using AMPure XP beads for PCR amplification and purification to obtain the final library. Preliminary quantification was then performed using the Qubit2.0 Fluorometer. The insert size of the library was detected using the Agilent 2,100 bioanalyzer. The effective concentration was accurately quantified by qRT-PCR to ensure the quality of the library. Once qualified, the library was sequenced on the Illumina Novaseq platform and generated 150 bp paired-end reads.

#### 2.4.3. Data analysis

Raw data in FASTQ format were initially processed using in-house Perl scripts. Clean data, which consisted of reads without adapters, N bases, and low-quality reads, was obtained from the raw data. All subsequent analyses were performed using the high-quality clean data. The reference genome and gene model annotation files were directly downloaded from the genome website. Rockhopper was utilized to identify novel genes, operons, transcription start sites, transcription termination sites, and cis-natural antisense transcripts. HTSeq v0.6.1 was used to quantify gene expression. Statistical analysis was accomplished with DESeq2 using a negative binomial distribution model. The resulting *p*-values were adjusted by adopting Benjamini and Hochberg’s approach for controlling the false discovery rate. Genes with an adjusted *p*-value <0.05 found by DESeq were determined as differentially expressed. Distinctions with *p* < 0.05 (*) or *p* < 0.01 (**) were deemed statistically noteworthy. Gene Ontology (GO) and Kyoto Encyclopedia of Genes and Genomes (KEGG) functional enrichment analyses were conducted to predict gene functions using clusterProfiler software.^2^ The experiment was conducted in triplicate under the same conditions.

### 2.5. Analytical methods

Lipid concentration was determined by ultraviolet spectrophotometry. After acidification with sulfuric acid, the culture medium was transferred into a separatory funnel. Petroleum ether was added, followed by shaking and standing stratification. The petroleum ether extract was then transferred into a volumetric flask, and petroleum ether was used for constant-volume adjustment. Absorbance was measured at 230 nm to calculate the lipid concentration. Cell growth (OD_600_) was evaluated using an ultraviolet spectrophotometer. TN content was determined by alkaline potassium persulfate digestion–ultraviolet spectrophotometry. NH_4_^+^-N content was determined by Nessler’s reagent spectrophotometry (Lin et al., 2014).

## 3. Results and discussion

### 3.1. Characterization of lipid-utilizing and denitrifying bacteria CYCN-C

**Figure 1:**
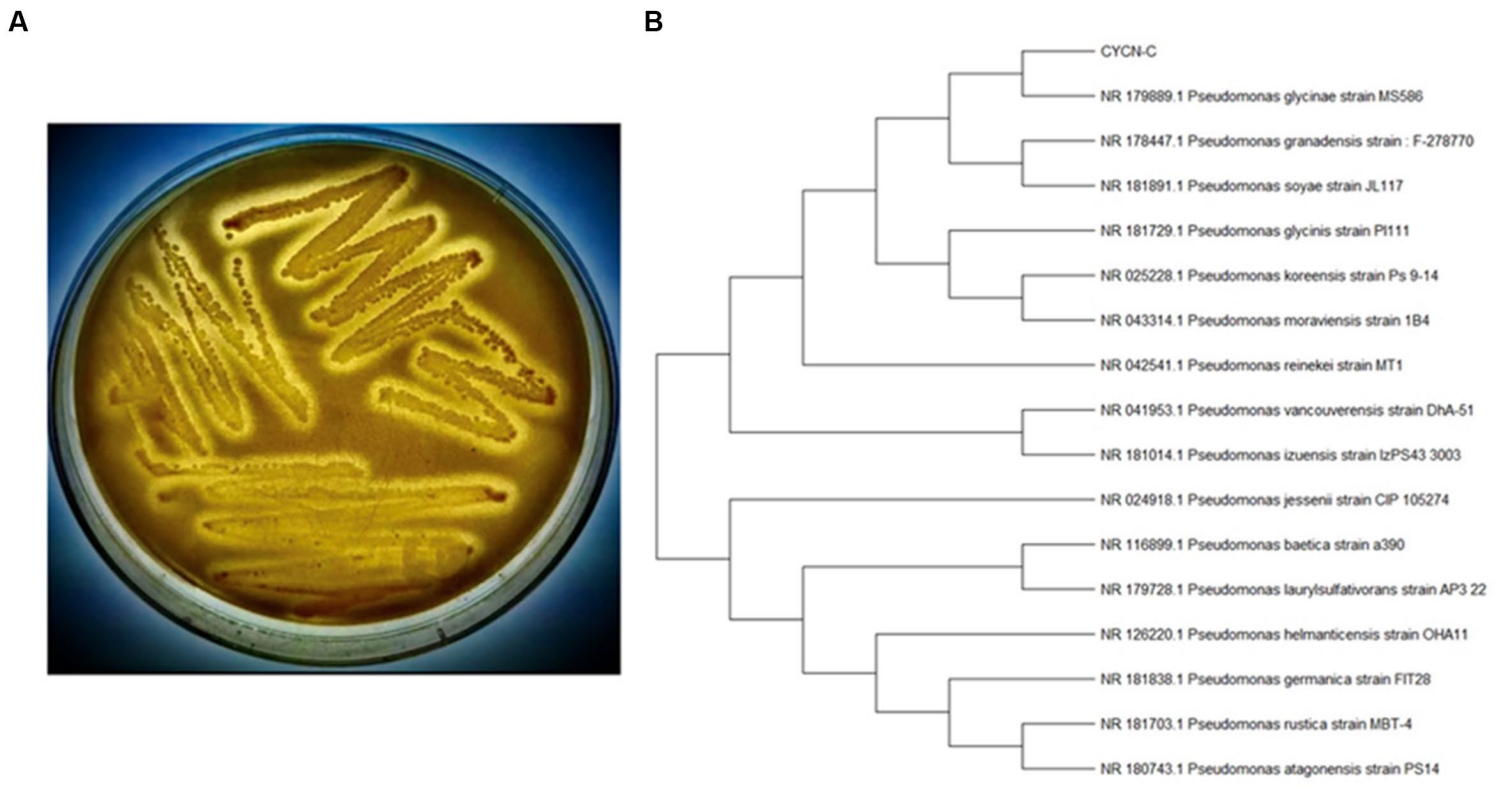
Lipolytic activity identification and phylogenetic analysis of strain CYCN-C. **(A)** Lipolytic activity assay of CYCN-C on lipase-production medium agar plates. **(B)** A phylogenetic tree was created based on the 16SrDNA gene sequences of strain CYCN-C.

Approximately 5,000 colonies of nitrogen-removing bacteria were obtained by performing a high-throughput screening on a kitchen sewer sewage sample. These clones were screened for lipolytic activity on lipase-production medium agar plates. One clone exhibiting lipolytic activity (Figure 1A) was selected out of the approximately 5,000 colonies and was further investigated. BLAST analysis of the 16S rDNA genes sequences suggested that this strain had sequence identity (approximately 99.7%) with *Pseudomonas glycinae*. Phylogenetic analysis of 16SrDNA genes showed that the isolated strain was affiliated with the genus *Pseudomonas* (Figure 1B).

### 3.2. Characterization of lipid-utilizing and denitrifying bacteria

**Figure 2:**
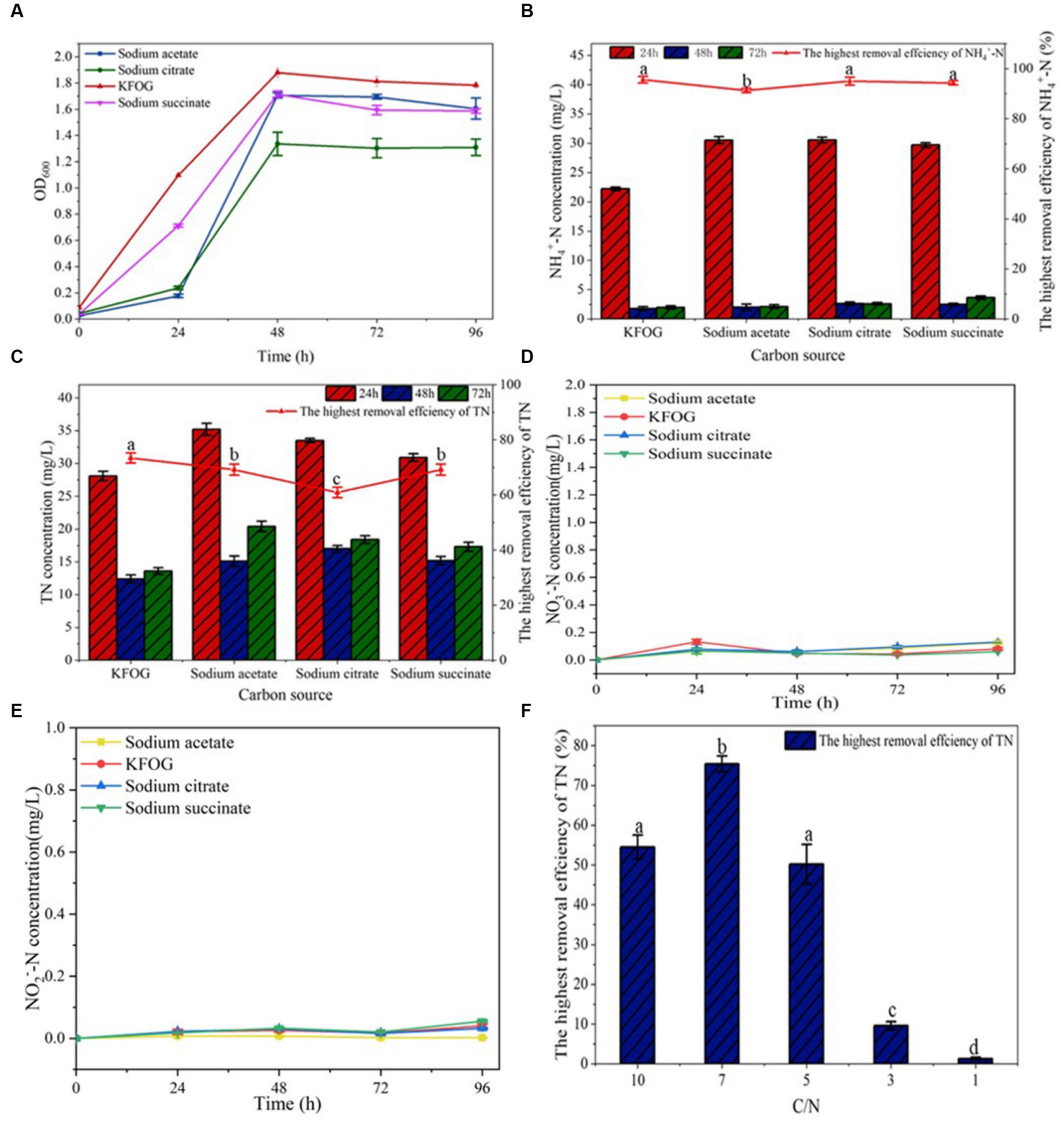
Verification of strain CYCN-C’s denitrification performance. **(A)** Growth curve under different carbon sources. **(B)** NH_4_^+^-N removal efficiency under different carbon sources. **(C)** TN removal efficiency under different carbon sources. **(D)** Variation of NO_3_^−^-N concentration in the medium of CYCN-C cultured with different carbon sources for 96 h. **(E)** Variation of NO_2_^−^-N concentration in the medium of CYCN-C cultured at different carbon sources for 96 h. **(F)** TN removal effect at different C/N ratios. [Different letters in the figure indicate significant differences between groups (*p* < 0.05)].

Carbon source is an important nutrient that influences the growth and metabolism of denitrifying bacteria. Carbon-source preferences exert different effects on the nitrogen-removal efficiency of denitrifying bacteria (Guo et al., 2013; Zeng et al., 2022). The growth rate of CYCN-C using KFOG as carbon source was significantly better than compared with that when using other carbon sources (Figure 2A). In the KFOG group, CYCN-C showed an exponential growth trend in a short time, and the maximum OD_600_ reached 1.88 at 48 h. Meanwhile, the OD_600_ values of the sodium acetate, sodium citrate, and sodium succinate groups reached 1.71, 1.34, and 1.72, respectively (Figure 2A). The nitrification and denitrification abilities of CYCN-C are shown Figures 2B,C. By using these carbon sources reached, CYCN-C reached the highest removal efficiency for NH_4_^+^-N and TN at 48 h. As shown in Figure 2B, the NH_4_^+^-N removal efficiency of CYCN-C with KFOG as the carbon source was the highest, reaching 95.6%. Conversely, the removal efficiency of CYCN-C with sodium acetate, sodium citrate, and sodium succinate as the carbon source were low at 91.3, 95 and 94.3%, respectively. Figure 2C shows that the highest TN removal rate of 73.4% was observed for CYCN-C with KFOG as the carbon source, and the lowest of 60.9% was found for CYCN-C with sodium citrate. Meanwhile, the removal rates of CYCN-C with sodium acetate and sodium succinate as the carbon source were high at 69.2 and 67.5%, respectively. These data further indicated that the presence of KFOG significantly enhanced the nitrification and denitrification abilities of CYCN-C. In addition, Figures 2D,E demonstrate that almost no accumulation of NO_3_^−^-N and NO_2_^−^-N was observed during the nitrogen removal, suggesting the suitability of CYCN-C for practical application in wastewater treatment.

The C/N ratio significantly affects the nitrogen -removal efficiency of microbes (Zhang et al., 2023). As shown Figure 2F, the highest TN removal efficiency (75.4%) was found at a C/N ratio of 7. Low TN removal efficiencies were observed at C/N ratios of 3 and 1. Conversely, the removal rates of C/N ratios at 10 and 5 were high at 54.5 and 50.2%, respectively. These results showed that CYCN-C had a moderate C/N requirement, and extremely low (1–3) and high (10) C/N ratios inhibited its denitrification ability. This finding was consistent with the previous report that too high or too low C/N can inhibit the metabolism of denitrifying bacterial, and consequently reduce the efficiency of nitrogen removal (Hu et al., 2023).

**Figure 3:**
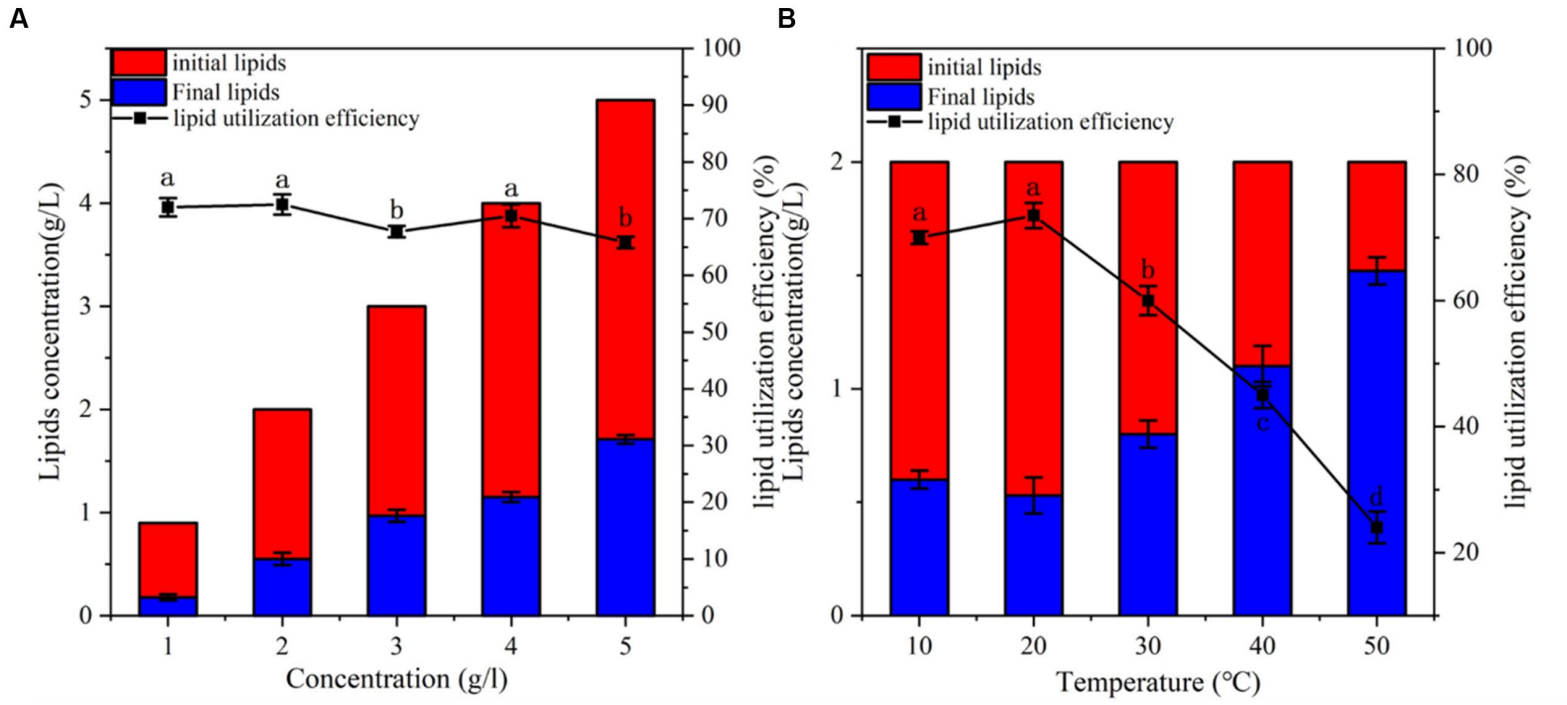
Verification of strain CYCN-C’s lipid -utilization efficiency. **(A)** Lipid -utilization efficiency at different concentrations. **(B)** Lipid -utilization efficiency at different temperatures. [Different letters in the figure indicate significant differences between groups (*p* < 0.05)].

Culture lipid concentration and temperature are important factors affecting the growth and metabolism of microorganisms. The effect of initial lipid concentration (1–5 g/L) on the lipid-utilization efficiency of the bacterium was also examined. The results showed that the highest lipid-utilization efficiency was achieved at a concentration of 2 g/L (Figure 3A). At the lipid concentration of 2 g/L, 72.5% of lipid was utilized within 2 days of incubation. The lipid utilization efficiency of CYCN-C was lower when the initial lipid concentration ranged 3–5 g/L (67.7, 70.5, and 65.8%, respectively). Oil can act as a carbon source to promote cell growth; however, at high concentrations, it blocks the aeration, thereby affecting the growth of bacteria and reducing their lipid -utilization efficiency (Ke et al., 2021). These phenomena were in line with the findings of this study.

The effects of temperature on the lipid-utilization efficiency of CYCN-C was evaluated (Figure 3A). The results demonstrated that with the increased in temperature, the lipid -utilization efficiency first increased and then slightly decreased before reaching the maximum of 73.5% at 20°C (Figure 3B). In addition, the lipid -utilization efficiency decreased significantly with the increasing temperature to over 30°C. After incubation at 30°C, 40°C, and 50°C for 2 days, the lipid-utilization efficiencies were 60, 45, and 24%, respectively. Under low-temperature conditions (10°C), the lipid-utilization rate of lipid was 70%, indicating that the strain was capable of efficient lipid utilization at low temperatures. These findings indicated the great potential of CYCN-C in future full-scale applications for low-temperature wastewater treatment.

CYCN-C can effectively utilize solidified oil as a carbon source to efficiently remove nitrogen from wastewater, especially for wastewater from the farmhouse and catering industries (Peng et al., 2014). Under low-temperature conditions, the grease present in wastewater tends to solidify and causes blockages and sedimentation in pipelines (Nimkande and Bafana, 2022). The impressive activity and nitrogen removal capacity of CYCN-C make it a promising option for the efficient treatment of oily wastewater.

### 3.3. Genes differential expression under different carbon sources

**Figure 4:**
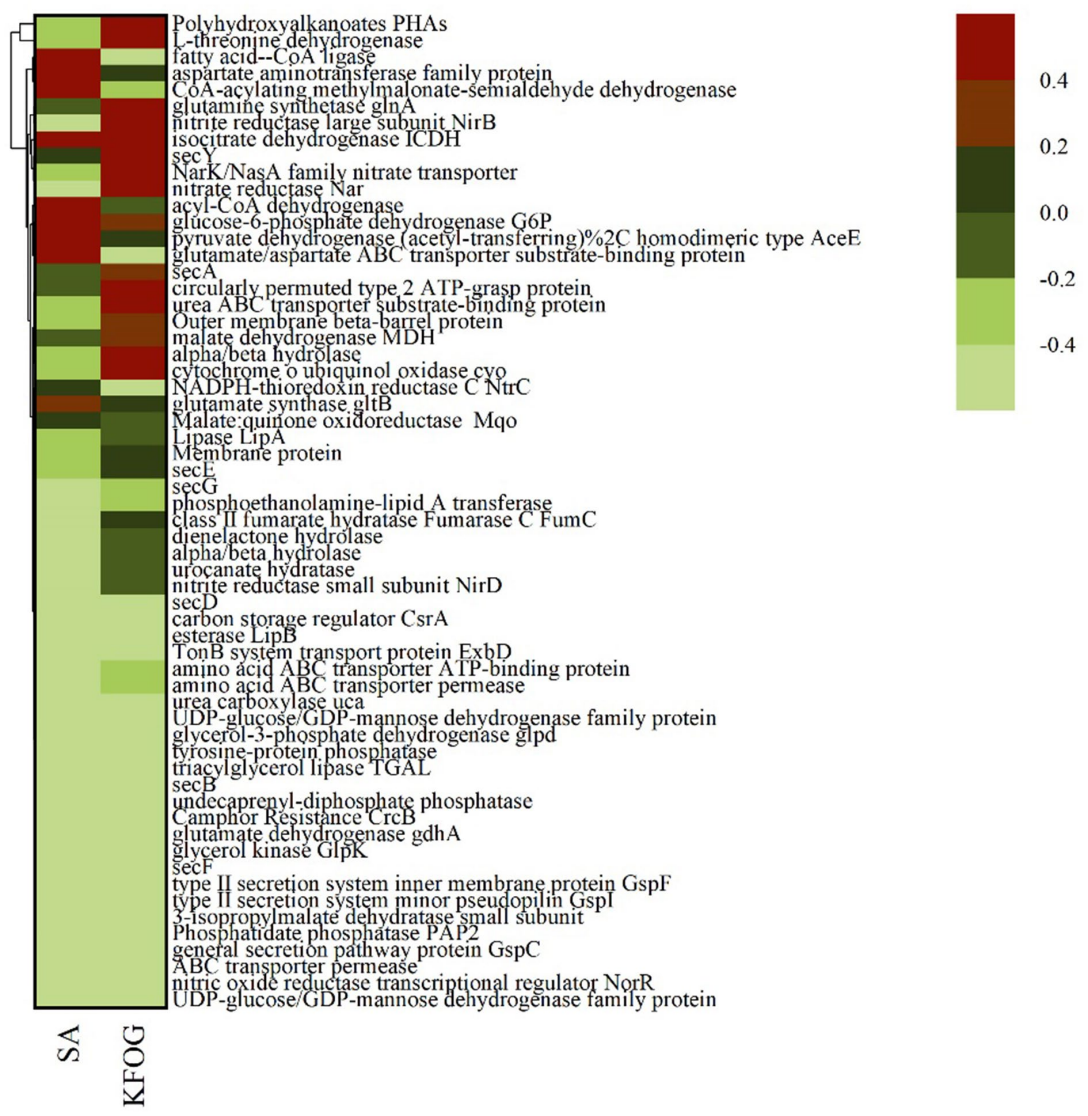
Expression levels of 60 differentially expressed genes under different carbon sources.

A total of 941 genes were differentially expressed between the sodium acetate group (SA) and KFOG group (KFOG) (threshold <= 0.005; |log2foldchange| > = 1.0). Among them, 567 genes were upregulated and 374 genes were downregulated in the KFOG group. Meanwhile, 60 significantly differentially expressed genes between the sodium acetate and KFOG groups were selected from the transcriptome sequencing results (Figure 4). These 60 genes included the energy metabolism-related genes, namely, polyhydroxyalkanoate gene (*PHA*), cytochrome o ubiquinol oxidase (*cyo*), and isocitrate dehydrogenase (*ICDH*); the lipid metabolism-related genes, namely, lipase gene (*lipA*), esterase gene (*lipB*), and glycerol kinase gene (*glpK*); and the nitrogen metabolism-related genes, namely, *NarK/NasA* family nitrate transporter, nitrate reductase (*nar*), and nitrite reductase (*nirB*). The results indicated that KFOG significantly affected the growth metabolism, lipid metabolism, and nitrogen metabolism of CYCN-C.

### 3.4. Effects of carbon sources on the expression levels of growth-metabolism-related genes

**Figure 5:**
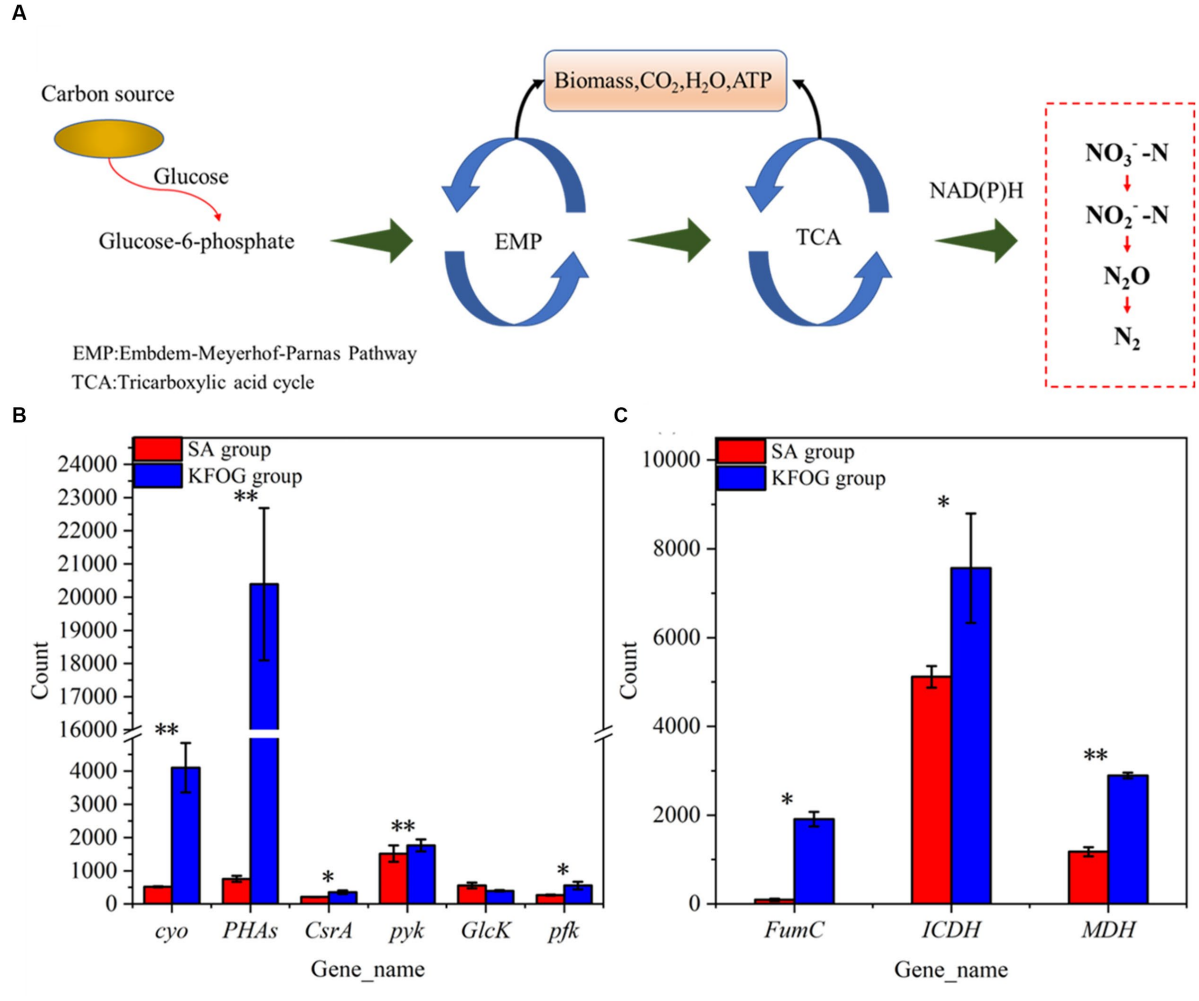
Carbon metabolism pathway and key genes. **(A)** Pathways of carbon metabolism in bacteria. **(B)** Genes involved in carbon metabolism. **(C)** Genes involved in the TCA cycle.

The cell growth and nitrogen removal of denitrifying bacteria affected by carbon metabolism (Figure 5A) (Yuan et al., 2022; Chen et al., 2023). The transcriptional levels of the differentially expressed genes involved in carbon metabolism were tested and compared between the KFOG and sodium acetate groups to investigate the effect of carbon source preference on carbon metabolism. The transcript level of cytochrome o ubiquinol oxidase gene (cyo) in the KFOG group was upregulated by 6.9-folds (Figure 5B). Bacteria are reported to exhibit carbon catabolite repression to regulate their carbon metabolism (Matar et al., 2017), cyo is one of the key enzymes involved in catabolic inhibition, and its transcription level varies according to growth conditions. In particular, cyo is highly expressed under preferred carbon sources (Dinamarca et al., 2003). Using a preferred carbon source is always advantageous for bacteria (Kleijn et al., 2010). This result indicated that KFOG was the preferred carbon source for CYCN-C over sodium acetate, which is consistent with previous results on carbon -source optimization. Furthermore, the transcript levels of carbon storage regulator protein gene (*CsrA*) and Polyhydroxyalkanoates gene (*PHAs*) in the KFOG group were upregulated by 0.7 and 26-folds, respectively. *PHAs* are intracellular energy storage components, involved in regulating the storage and utilization of carbon sources, improving the functional genes of the denitrification system, and facilitating the expression of nitrate reductase (Wu et al., 2021). *CsrA* plays an important role in the regulation of various physiological processes, such as carbon metabolism, acetic acid metabolism, and biofilm formation, and has a positive regulatory effect on glycolysis (Timmermans and Van Melderen, 2010). Glucokinase gene (*GlcK*), phosphofructokinase gene (*pfk*), and pyruvate kinase gene (*pyk*) involved in glycolysis (Embden–Meyerhof–Parnas) were detected in this study. The transcript levels of *pyk* and *pfk* in the KFOG group were upregulated by 0.2 and 1.1-folds, respectively. Carbon sources play a crucial role in providing electron and energy for cell metabolism through glycolysis and are advantageous for cell growth and nitrogen removal (Padhi and Maiti, 2017; Yang et al., 2021). These results showed that KFOG provided additional energy and electrons for cell growth and promoted carbon and nitrogen metabolism and was also conducive to bacterial growth and nitrogen removal.

As shown in Figure 5C, the key enzyme genes (*FumC*, *ICDH*, and *MDH*) involved in the tricarboxylic acid (TCA) cycle were detected. Compared with those in the sodium acetate group, the expression levels of *FumC*, *ICDH*, and *MDH* in the KFOG group were upregulated by 19, 0.5, and 1.4-folds, respectively. The TCA cycle is the core metabolism pathway of various nutrients in organisms. The TCA cycle of denitrifying bacteria is very active (Zhang H. et al., 2020; Meng et al., 2023). In the TCA cycle, cytosolic fumarase (*FumC*) catalyzes the conversion of fumaric acid to L-malic acid. Malate dehydrogenase (*MDH*) catalyzes malic acid to form oxaloacetic acid, and its activity reflects the activity of the TCA cycle. Isocitrate dehydrogenase (*ICDH*) is an important rate-limiting enzyme in the TCA cycle. It can regulate isocitrate flow into the TCA cycle and catalyze isocitrate to produce α-ketoglutaric acid (Zhou S. et al., 2023). The results indicated that the TCA cycle of CYCN-C in lipid was highly active. The capacity of nitrogen removing bacteria to utilize carbon sources is reportedly affected by many factors, but their preferred carbon source directly participates in the TCA cycle and provides additional electrons for increased energy and for their own growth, proliferation, and nitrogen removal (Jia et al., 2019). Thus, CYCN-C can better utilize KFOG as a carbon source than sodium acetate.

### 3.5. Effects of carbon sources on the expression level of lipid-metabolism-related genes

**Figure 6:**
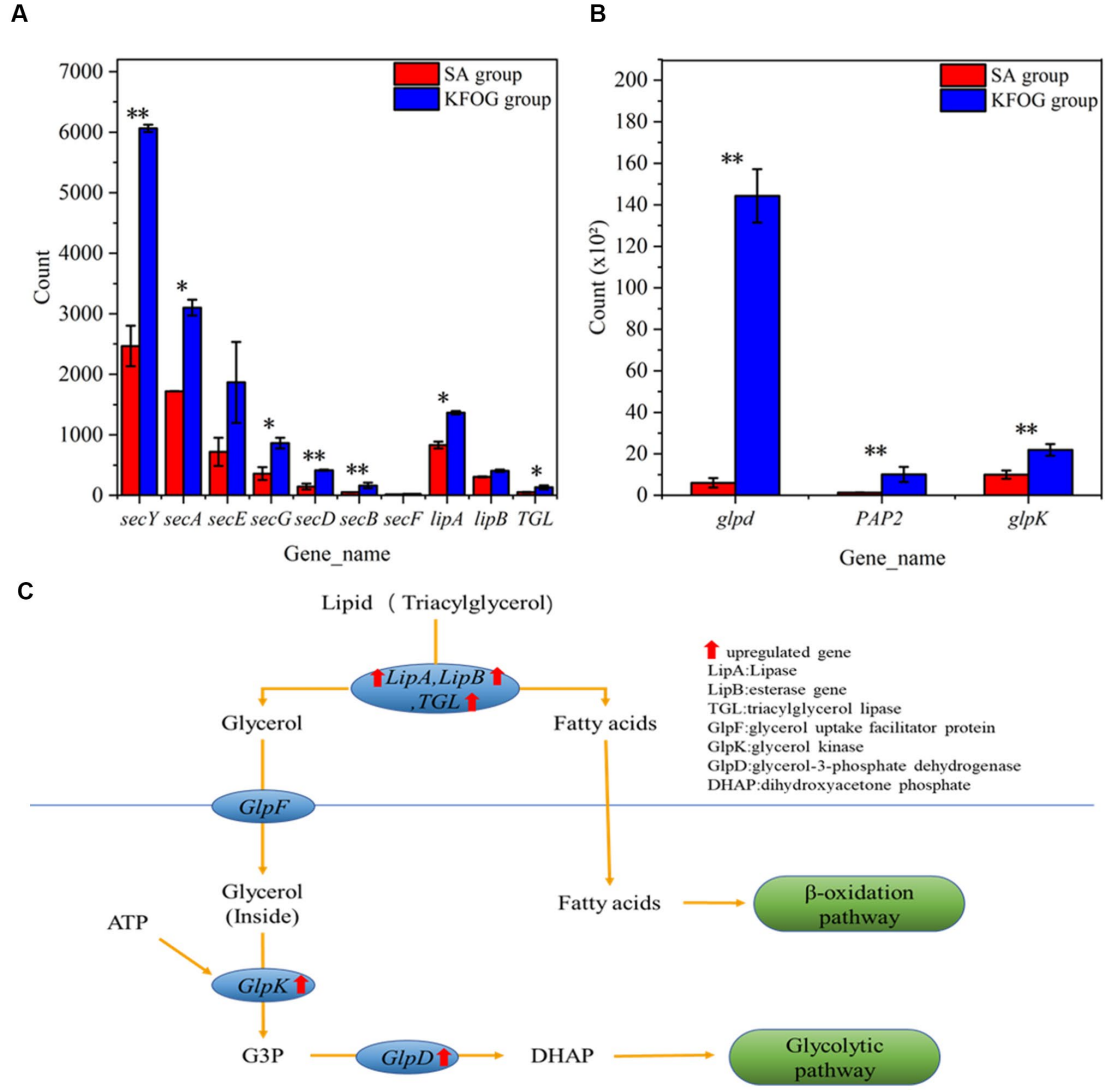
Genes related to lipid metabolism. **(A)** Genes related to lipase transport and expression. **(B)** Genes related to glycerol metabolic. **(C)** Pathways of lipid metabolism in bacteria.

Lipase gene (*lipA*), esterase gene (*lipB*), triacylglycerol lipase gene (*TGL*), and *Sec* family genes (*secY*, *secA*, *secE*, *secG*, *secD*, *secB*, and *secF*) were detected to further investigate the lipid metabolism pathway of CYCN-C as shown in Figure 6A. The results showed that the transcription levels of *lipA, TGL, lipB*, and *Sec* genes in the KFOG group sharply increased. In particular, the expression levels of *lipA*, *lipB*, and *TGL* were upregulated by 0.6, 0.4, and 1.5-folds, respectively. Lipase and esterase are enzymes that catalyze the hydrolysis of triglycerides (Eggert et al., 2001) and determine the utilization efficiency of oil carbon sources for cell growth. Lipases in *Pseudomonas* are secretions across the inner membrane via the Sec-machinery (Rosenau and Jaeger, 2000). The transcription results explained the efficient lipid removal of CYCN-C strain. In addition, the genes related to lipolytic enzymes were significantly upregulated in the presence of KFOG. This result was consistent with previous reports, stating that the gene expression of lipases was inducible by substrate, and significantly increased in the presence of grease carbon source (Poblete-Castro et al., 2020). This finding indicated that CYCN-C played a positive role in growth and metabolic activities during lipid use and degradation.

In waste-oil biodegradation, the lipids in water are hydrolyzed by lipase to produce fatty acids and glycerol. Glycerol is then catabolized and converted through the glycerol metabolic pathway. As shown in Figure 6B, glycerol-3-phosphate dehydrogenase (*glpD*), glycerol kinase (*glpK*), and phosphatidate phosphatase (*PAP2*) genes were detected and found to be upregulated in the KFOG group by 21.5, 1.3, and 6.5-folds, respectively. As shown Figure 6C, glycerol was converted to glycerol-3-phosphate (G3P) by *glpK*. Afterward *glpD* catalyzed G3P to dihydroxyacetone phosphate and entered the glycolytic pathway. A positive correlation might exists between *glpK* expression and glycerol metabolism (Tang et al., 2023). These results showed that the glycerol metabolic pathway in CYCN-C was hyperactive in the KFOG group, providing abundant energy for cell growth and metabolism. Furthermore, PAP enzyme played an important role in the generation or degradation of lipid (Carman and Han, 2006). In the KFOG group, *PAP2* was significantly upregulated, which had a positive regulatory effect on lipid metabolism (Hsieh et al., 2012).

### 3.6. Effects of carbon sources on the expression level of nitrogen-metabolism-related genes

**Figure 7:**
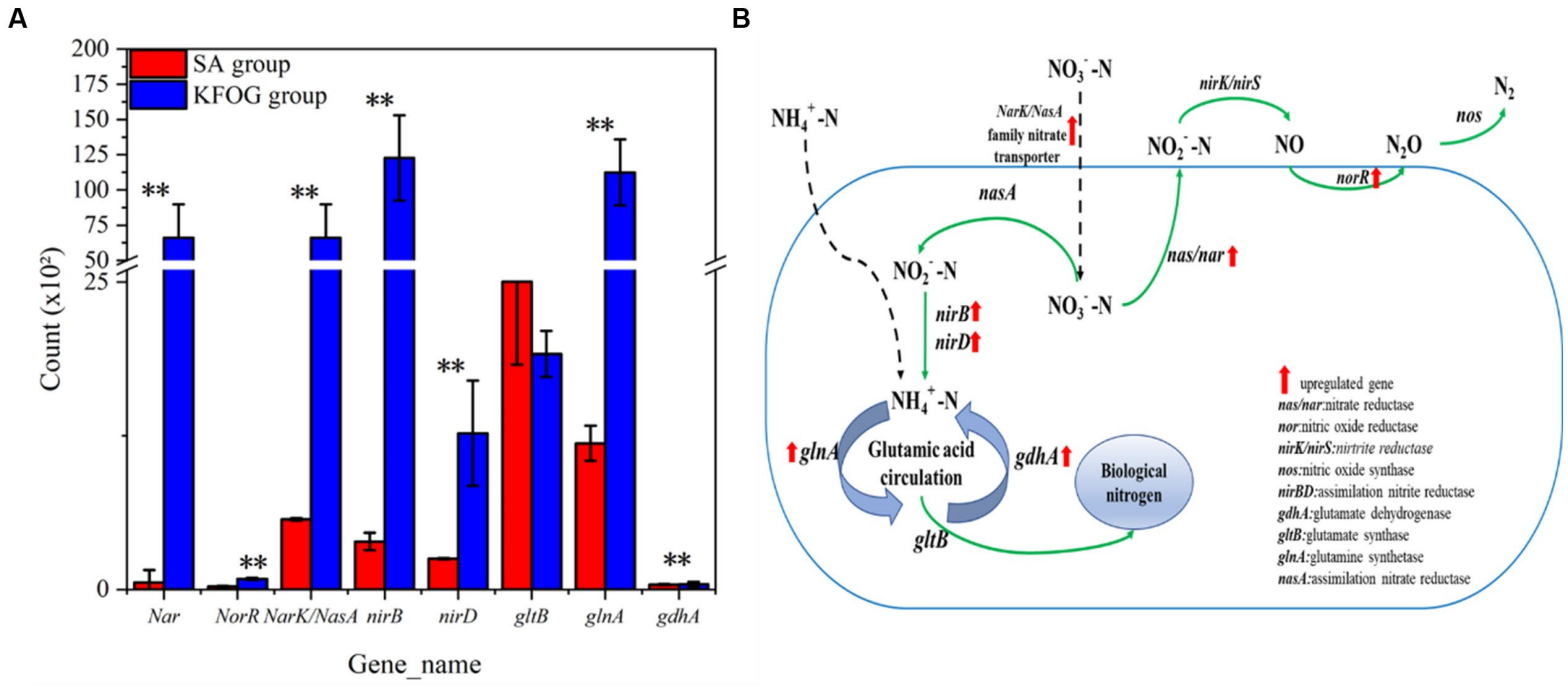
Genes related to nitrogen metabolism. **(A)** Expression of nitrogen-metabolism-related gene. **(B)** Nitrogen metabolism pathway and key genes.

CYCN-C with KFOG or sodium acetate as the carbon source was analyzed by transcriptome sequencing to further investigate the influence of grease carbon sources on the biological nitrogen removal. The nitrogen -removal pathways and the expression of related genes under different carbon sources were revealed based on the identified functional genes (Figure 7A) involved in nitrogen metabolism, including nitrate reductase (*nar*), nitrite reductase (*nirB* and *nirD*), *NarK*/*NasA* family nitrate transporter, nitric oxide reductase transcriptional regulator (*norR*), glutamate dehydrogenase (*gdhA*), glutamine synthetase (*glnA*), and glutamate synthase (*gltB*).

In the KFOG group, genes *norR*, *NarK/NasA* family nitrate transporter, and *nar* were upregulated by 3, 10.6, and 115-folds, respectively. In addition, *glnA* and nitrite reductase related functional genes *nirB* and *nirD* were upregulated by 8, 30, and 49-folds, respectively. In denitrifying bacteria, the *NarK/NasA* family nitrate transporter belongs to the major facilitator superfamily that transports nitrate or nitrite across membranes. Inorganic nitrogen was primarily transformed into organic nitrogen and N_2_ through assimilation and dissimilation (Figure 7B). In the dissimilation pathway, NO_3_^−^-N was converted into NO_2_^−^-N by *nar*, and the nitrite reductase converted NO_2_^−^-N into NO. *NorR* and nitrous oxide reductase (*nos*) converted NO into N_2_. Notably, the *nos*, *nirS/K*, and *nap* genes were not detected in the CYCN-C genome, and the functions of the three nitrate reductase enzymes *nas*, *nar*, and *nap*, in nitrification and denitrification are not absolute (Zhang et al., 2019; Zhang M. et al., 2020). It has been reported that many denitrifying bacteria cannot carry out the whole denitrification process, just like CYCN-C. The key enzyme genes in the nitrogen pathway were significantly upregulated, and KFOG as the carbon source enhanced the dissimilated denitrification ability of CYCN-C. Grease as a carbon source could provide additional electron donors for nitrogen metabolism, thereby increasing the nitrogen -removal efficiency (He et al., 2015). Meanwhile, the assimilation pathway was maintained by nitrate reductase (*nasA*) and nitrite reductase (*nirB/nirD*), which reduced NO_3_^−^ − N to NO_3_^−^ − N and NO_3_^−^ − N to NH_4_^+^-N. Under the catalysis of *gdhA, glnA*, and *gltB* genes, NH_4_^+^-N was converted into biological nitrogen for cell growth and metabolism (Zhou X. et al., 2023). Thus, the assimilation gene was significantly upregulated, indicating that the assimilation pathway became highly active when KFOG served as the carbon source. Ammonia-assimilating microorganisms exist extensively in nature and are conducive to assimilation in the presence of sufficient carbon sources (Xu et al., 2023).

In summary, CYCN-C may differ from typical denitrifying bacteria. We speculated that during nitrogen metabolism, CYCN-C assimilated a part of inorganic nitrogen into biological nitrogen, and another part of inorganic nitrogen was converted into N_2_ through the dissimilation pathway. When KFOG served as the carbon source, the genes related to nitrogen metabolism were significantly upregulated and the activity of nitrogen metabolism was increased. The efficiency of denitrification with KFOG as carbon source was higher than that with sodium acetate.

## 4. Conclusion

CYCN-C, a strain of *Pseudomonas* with lipid-degrading activity and high nitrogen-removal efficiency was isolated and characterized. The TN removal efficiency can reach 75.4% with KFOG as the carbon source. The effects of KFOG as the carbon source on the growth metabolism, lipid metabolism, and nitrogen metabolism of CYCN-C were analyzed at the transcriptomic level. The key enzyme genes in the lipid-utilization pathway and nitrogen metabolism were identified. This study verified the feasibility of waste lipid as an *in-situ* carbon source for wastewater treatment and provided novel insights into the metabolic mechanism underlying the simultaneous lipid utilization and denitrification of this bacterium.

## Data availability statement

The original contributions presented in the study are included in the article/supplementary material, further inquiries can be directed to the corresponding authors.

## Author contributions

YT: Data curation, Formal analysis, Investigation, Methodology, Writing – original draft. YL: Data curation, Formal analysis, Investigation, Methodology, Writing – review & editing. WQ: Data curation, Formal analysis, Methodology, Writing – review & editing. SW: Formal analysis, Investigation, Resources, Writing – review & editing. WX: Formal analysis, Investigation, Methodology, Writing – review & editing. PJ: Conceptualization, Data curation, Formal analysis, Methodology, Supervision, Writing – review & editing. ZZ: Conceptualization, Project administration, Writing – review & editing.

## Footnotes

^1^ blast.ncbi.nlm.nih.gov

^2^ http://www.broadinstitute.org/gsea/index.jsp
